# Dynamic high-sensitivity troponin elevations in atrial fibrillation patients might not be associated with significant coronary artery disease

**DOI:** 10.1186/s12872-017-0601-7

**Published:** 2017-06-27

**Authors:** Johan Thelin, Olle Melander

**Affiliations:** 1 Department of Clinical Sciences, Lund University, Lund, Sweden; 2 Present address: Department of Internal Medicine, Skåne University Hospital, Akutmottagningen, Klinikgatan 15, 2285 Lund, Sweden; 3 Present address: CRC, Jan Waldenströms gata 35, hus 91, plan 12, 20502 Malmö, Sweden

**Keywords:** Type 2 myocardial infarction, Atrial fibrillation, High sensitivity troponin, Coronary artery disease, Tachycardia

## Abstract

**Background:**

Since the introduction of high-sensitivity troponin assays a greater proportion of atrial fibrillation (AF) patients present with dynamic troponin elevations. We hypothesize that significant coronary artery disease (CAD) causes relative ischemia in the setting of a rapid heart rate resulting in dynamic troponin elevation. The aim of this study was to examine if patients without known CAD who present with AF, tachycardia and dynamic high-sensitivity troponin T (hsTnT) change have an increased risk of cardiac events.

**Methods:**

We retrospectively included AF patients presenting with tachycardia during one year. The primary endpoint was acute coronary syndrome, revascularization or death due to ischemic heart disease during 30 months follow-up.

**Results:**

Five hundred twenty-two patients without known CAD were included, 300 (57%) had normal hsTnT and 49 (9.5%) had dynamic hsTnT elevation. During follow-up 12 (4%) patients with normal hsTnT reached the primary endpoint and a total of 14 (4.7%) patients died. In the group with dynamic hsTnT the results were 4 (8.2%) and 12 (25%) respectively. The age-adjusted hazard ratio (HR) for the primary endpoint in patients with dynamic hsTnT was 1.9 (95% CI: 0.6 to 6.2; *p* = 0.28) and for all-cause mortality 3.8 (95% CI: 1.7 to 8.5; *p* = 0.001).

**Conclusions:**

Dynamic hsTnT elevation in connection with AF might not be associated with any major increased risk of coronary events, but indicates increased all-cause mortality.

**Electronic supplementary material:**

The online version of this article (doi:10.1186/s12872-017-0601-7) contains supplementary material, which is available to authorized users.

## Background

Atrial fibrillation (AF) is the most common supraventricular arrhythmia encountered in clinical practice [1]. The cause of emergency department (ED) visits is often symptoms like palpitations, chest pain, dyspnea, fatigue or dizziness and these complaints are frequently attributed to a rapid heart rate [2–4]. Due to the diffuse symptomatology, cardiac troponins are commonly analysed, often showing elevated values whose significance and causes in many cases are unclear [3, 5, 6].

The population of AF patients with elevated cardiac troponins has increased in size since the introduction of high sensitivity troponin (hsTn) assays, because of lower decision cut-offs, and in clinical practice we have noted that an increasing proportion of AF patients with rapid ventricular response (RVR) have minor troponin elevations with a significant rising and/or falling pattern (i.e. significant dynamic change) [7–10]. Many of these patients also show clinical evidence of acute myocardial ischemia (i.e. chest pain or new electrocardiography (ECG) changes) and have according to current guidelines suffered a type 2 myocardial infarction (MI) [11–13]. Type 2 MI is by definition caused by ischemia due to an imbalance between oxygen supply and demand.

Although the research on type 2 MI is limited, it has been reported that approximately 20–25% of the cases are caused by tachyarrhythmias and that type 2 MI is associated with a poor prognosis [12, 14]. Previous studies have also shown that elevated troponin (eventual dynamic change was not specified) in AF patients is an independent risk marker for cardiovascular morbidity and mortality [15–18] and that AF patients might have an increased prevalence of subclinical coronary artery disease (CAD) [19, 20]. The reason why only some AF patients with RVR suffer a type 2 MI and how to best manage dynamic troponin elevations in these patients is unknown.

We hypothesize that AF patients suffering type 2 MI might have a significant unknown CAD, which in the setting of a rapid heart rate with increased myocardial oxygen demand causes relative ischemia and subsequent troponin release from cardiomyocytes. If this is the case, urgent cardiac evaluation and intensive prophylactic measures might be indicated in this population.

The aim of this study was to examine if patients without known CAD who present with AF, RVR (heart rate ≥ 110 beats/min) and dynamic troponin elevation have an increased risk of acute coronary syndrome (ACS) or death due to ischemic heart disease during follow-up.

Our secondary aims were: (1) To describe the incidence of, and patient characteristics associated with, dynamic troponin elevation in AF patients with RVR. (2) To extend our primary analysis to patients with known CAD and to patients presenting with troponin elevation without significant dynamic change.

## Methods

### Study design

The study included patients at two different sites, the Skåne University Hospital in Lund and the Skåne University Hospital in Malmö, Sweden, which combined serve as the primary hospitals for approximately 650,000 inhabitants. All patients admitted to the hospital observation unit receiving the *International Classification of Diseases,* version 10 (ICD-10), code I48 (atrial fibrillation or atrial flutter) as primary diagnosis during one year were identified by searching our local administrative database (PASIS). The dates when the two hospitals introduced high sensitivity troponin T (hsTnT) analysis were chosen as starting dates, February 22th 2010 in Lund and May 3rd 2011 in Malmö. The follow-up period was 30 months for each patient.

The study was conducted according to the principles of the Declaration of Helsinki and approved by the Regional Ethics Committee in Lund, Lund University (registry number 2014/453). The Regional Ethics Committee did not request an informed consent.

### Data collection and exclusion criteria

Data including admission ECG, laboratory results, clinical and background variables was extracted from medical records according to a predefined protocol. If the patient had more than one care episode during the year of inclusion, only the first admission was included for analysis.

**Fig. 1:**
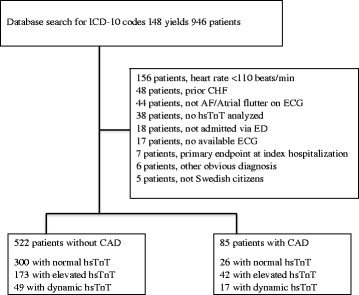
Chart view of study patients. *CHF* chronic heart failure, *AF* atrial fibrillation, *hsTnT* high sensitivity troponin T, *ED* emergency department, *ECG* electrocardiography, *CAD* coronary artery disease

Exclusion criteria included (1) patients not admitted via the ED, (2) patients with another obvious primary diagnosis, (3) patients that were not Swedish citizens (no follow-up available), (4) patients with no available ECG or ECG with other rhythm than AF/atrial flutter, (5) patients with heart rate on admission ECG < 110 beats/min, (6) patients with no hsTnT analysed, (7) patients with chronic heart failure or (8) patients who reached the primary endpoint (see below) in the hospital stay when they were included (as the possible troponin elevation in these patients is most likely caused by a type 1 MI) (Fig. 1).

Follow-up data were obtained from the Swedish National Board of Health and Welfare records. ICD-10 codes for diagnoses and codes for procedures were acquired from the National Patient Register that holds all relevant Swedish inpatient and outpatient visits (http://www.socialstyrelsen.se/register/halsodataregister/patientregistret/inenglish) and ICD-10 codes for cause of death were acquired from the National Cause of Death Register (http://www.socialstyrelsen.se/register/dodsorsaksregistret).

### Definitions and troponin T analysis

Significant CAD was defined as prior percutaneous coronary intervention (PCI), coronary artery bypass grafting (CABG) or a > 50% stenosis on coronary angiography. Chronic heart failure (CHF) was defined as having prior echocardiography showing ejection fraction (EF) ≤45%, moderate to severe valvular disease, hypertrophic cardiomyopathy or dilated cardiomyopathy. Chest pain was defined as any chest discomfort, beyond palpitations, described as more than mild. Hypertension and hyperlipidemia were defined as ongoing medication for each condition. Data on diabetes, prior AF or atrial flutter and prior stroke or transient ischemic attack (TIA) were collected from medical records. Significant ST depression was defined according to current guidelines [11, 21].

Type 2 myocardial infarction was defined according to current guidelines [11]. Thus, significant rising or falling hsTnT values and clinical evidence of acute myocardial ischemia (i.e. chest pain and/or new electrocardiography (ECG) changes) were required. The myocardial ischemia also had to be considered caused by an imbalance of myocardial blood supply and demand rather than by plaque rupture and/or thrombosis.

The hsTnT method used, previously described in [22, 23], was Roche hsTnT, with a Limit of detection (LoD) of 5 ng/L, normal range 5–14 ng/L and a troponin T value of >14 ng/L (99th percentile) was considered elevated [24]. Significant rising or falling hsTnT values (i.e. significant dynamic change) were defined as (1) an initial normal hsTnT value in combination with a ≥ 50% rise to a value above the 99th percentile, (2) an initial slightly elevated (between 15 and 50 ng/L) value in combination with a ≥ 50% change or (3) an initial value of >50 ng/L in combination with a ≥ 20% change [8].

Patients were divided into three different groups depending on troponin values, normal troponin without dynamic change (normal hsTnT), elevated troponin (elevated hsTnT) (without significant dynamic change or with only one troponin sample available) or elevated troponin with significant dynamic change (dynamic hsTnT).

### Endpoints

The primary endpoint was defined as ACS (unstable angina (UA) (ICD-10 code I200) or acute myocardial infarction (AMI) (ICD-10 codes I21)) as primary diagnosis, revascularization (ICD-10 codes FN) or death due to ischemic heart disease (ICD-10 codes I21–25). ICD-10 codes I21-I24 covers death due to AMI but also deaths with the primary cause given as ICD-10 code I25, chronic ischemic heart disease, was included in the primary endpoint. If UA or AMI were given as secondary diagnoses, they were not included in the primary endpoint.

Secondary endpoints were: (1) cardiovascular death, defined as all I codes in ICD-10, (2) all-cause mortality.

### Statistical analysis

Continuous variables are presented as medians with the interquartile range and compared with the Mann-Whitney test. Categorical variables are presented as number and percentages and compared using the Pearson chi-square test or Fisher’s exact test if the expected count was <5.

Cox proportional hazard regression analysis was used to determine the relationship between hsTnT groups and the primary and secondary endpoints. Due to the low number of events we were only able to adjust for age in the multivariable analyses. The results are presented as hazard ratios (HR) with 95% confidence intervals (CI). Kaplan Meier plots were used to illustrate the timing of events and the Breslow test was used for statistical comparison.

Multivariable logistic regression analysis with dynamic troponin elevation as the dependent variable and presence of known CAD and other clinically relevant background factors as covariables were performed for all patients, for patients with normal hsTnT or dynamic hsTnT and for patients with elevated hsTnT or dynamic hsTnT. The results were presented as odds ratios (OR) with 95% CI.

Because of the limited previous studies in this area, we were not able to define the expected effect size for presence in dynamic hsTnT in relation to the primary endpoint and thus abstained from doing a power calculation. Instead we performed a post hoc power calculation in order to test which effect size we could expect if the null hypothesis was to be rejected, given the numbers we had access to.

All tests were two tailed. Data management and statistical analysis were performed using IBM SPSS Statistics, version 22.

## Results

As shown in Fig. 1, a total of 946 patients with the primary diagnosis of AF or atrial flutter were identified. Of these 339 were excluded resulting in 607 included patients and 40 (7%) reached the primary endpoint. Approximately 85% had AF and the remaining 15% had atrial flutter at presentation. Seven patients reached the primary endpoint at the index hospitalisation. All of these patients were transferred from the observation unit to a cardiac/internal medicine department were their primary ICD-10 diagnosis was changed from I48 (atrial fibrillation/flutter) to I21 (acute myocardial infarction).

The 522 patients without CAD were available for the primary analysis. Three hundred (57,5%) had normal hsTnT, 173 (33%) had elevated hsTnT without dynamic change (52 patients had only one hsTnT sample analyzed) and 49 (9.5%) had a significant dynamic hsTnT change. Eighty-five patients had known CAD and were available for secondary analysis.

Background characteristics, clinical variables at presentation and outcomes are shown in Table 1 for patients without CAD and in Additional file 1: Table S1 for patients with CAD. Patients without known CAD with dynamic hsTnT were older, had a higher prevalence of diabetes and prior stroke/TIA, higher heart rates on ECG at presentation, a higher prevalence of significant ST depression on ECG and had more frequently chest pain, compared to patients with normal hsTnT.

**Table 1:** Baseline characteristics, presentation and outcomes in the study population without CAD according to troponin subgroups

	Normal hsTnT (*n* = 300)	Elevated hsTnT (*n* = 173)	Dynamic hsTnT (*n* = 49)	*p*-value^a^
Age (years)	67 (59–74)	77 (69–85)	75 (68–83)	<0.001
Male sex	149 (50%)	85 (49%)	21 (43%)	0.44
Current smoking	41/266 (15%)	20/153 (13%)	5/45 (11%)	0.65
Hypertension	133 (44%)	102 (59%)	26 (53%)	0.27
Hyperlipidemia	59 (20%)	42 (24%)	10 (20%)	0.85
Diabetes	21 (7%)	28 (16%)	8 (16%)	0.05
Prior stroke/TIA	26 (9%)	26 (15%)	10 (20%)	0.01
Prior AF/atrial flutter	160 (53%)	88 (51%)	20 (41%)	0.12
CHA_2_DS_2_VASc				
≤1	131 (44%)	29 (17%)	5 (10%)	<0.001
2	64 (21%)	35 (20%)	10 (20%)	0.88
3	45 (15%)	42 (24%)	15 (31%)	0.007
4	37 (12%)	46 (27%)	12 (25%)	0.02
≥5	23 (8%)	21 (12%)	7 (14%)	0.16
Systolic BP (mmHg)	140 (125–155)	135 (120–150)	140 (124–157)	1.0
Heart rate (beats/min)	132 (119–146)	138 (126–155)	137 (127–151)	0.02
Hemoglobin (g/L)	148 (138–158)	135 (123–152)	141 (133–149)	0.003
Creatinine (μg/L)	83 (69–95)	96 (76–121)	88 (72–101)	0.16
CRP (mg/L)	2.1 (0.9–5.3)	6.0 (1.8–27)	3.1 (1.1–12.5)	0.14
Peak hsTnT (ng/L)	6 (4–10)	25 (19–36)	56 (25–101)	<0.001
Chest pain	67 (22%)	41 (24%)	20 (41%)	0.006
ST depression	48 (16%)	25 (15%)	17 (35%)	0.007
Atrial fibrillation	254 (85%)	147 (85%)	43 (88%)	0.67
CVD death	8 (2.7%)	28 (16%)	5 (10%)	0.02
All cause mortality	14 (4.7%)	50 (29%)	12 (25%)	0.001
Primary endpoint	12 (4%)	10 (5.8%)	4 (8.2%)	0.17

Data are presented as n (%) of patients or median and 25th–75th interquartile range for continous variables. Variables are defined in methods

*CAD* coronary artery disease, *hsTnT* high sensitivity troponin T, *TIA* transient ischemic attack, *AF* atrial fibrillation, *BP* blood pressure, *CVD* cardiovascular death

^a^normal hsTnT compared to dynamic hsTnT

### Primary and secondary endpoints in patients without known CAD (*n* = 522)

**Fig. 2:**
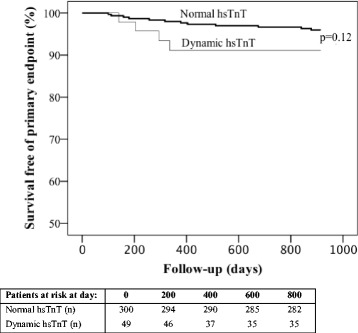
Kaplan Meier plot of survival free of primary end point in relation to normal troponin vs. dynamic hsTnT elevation in patients without known CAD. *p*-value by Breslow test, *CAD* coronary artery disease, *hsTnT* high sensitivity troponin T

Four patients (8.2%) with dynamic hsTnT (*n* = 49) and 12 patients (4%) with normal hsTnT (*n* = 300) reached the primary endpoint during follow-up (Table 1). The Kaplan Meier plot of survival free of primary endpoint in the two groups is illustrated in Fig. 2. Univariable cox proportional hazard regression analyses with hazard ratios for clinically relevant factors to predict the primary endpoint (16 events) is shown in Table 2. The age-adjusted HR for the primary endpoint in patients with dynamic hsTnT compared to patients with normal hsTnT was 1.9 (95% CI: 0.6 to 6.2; *p* = 0.28) (Table 2) and adjusted for age dynamic hsTnT versus normal hsTnT was associated with an increased risk of all-cause mortality, HR 3.8 (95% CI: 1.7 to 8.5; *p* = 0.001).

**Table 2:** Hazard ratios for the primary endpoint in patients without CAD. Patients with stationary elevated hsTnT are excluded

	Univariable analysis		Multivariable analysis^a^	
*n* = 349	HR (95% CI)	*p*-value	HR (95% CI)	*p*-value
Dynamic hsTnT	2.4 (0.76–7.3)	0.14	1.9 (0.59–6.2)	0.28
Age (years)	1.03 (0.99–1.08)	0.15	1.03 (0.98–1.07)	0.25
Male sex	1.4 (0.51–3.7)	0.39		
Diabetes	0.75 (0.10–5.7)	0.78		
Prior Stroke/TIA	0.60 (0.08–4.6)	0.63		
Heart rate (beats/min)	1.02 (0.99–1.04)	0.25		
Hemoglobin (g/L)	1.00 (0.97–1.04)	0.92		
Chest pain	1.0 (0.32–3.1)	0.99		
ST depression on ECG	1.5 (0.49–4.7)	0.48		

*HR* hazard ratio, *CAD* coronary artery disease, *hsTnT* high sensitivity troponin T, *CI* confidence interval, *ECG* electrocardiography

^a^Adjustments were made for dynamic hsTnT and age

In an age-adjusted Cox regression model we did not observe any increased risk of the primary endpoint (22 events) in patients with elevated hsTnT without dynamic change (*n* = 173) compared to patients with normal hsTnT (HR 1.3; 95% CI 0.5–3.1; *p* = 0.61), however elevated hsTnT without dynamic change adjusted for age was related to an increased risk of all-cause mortality (HR 4.3; 95% CI 2.3–8.0; *p* < 0.001).

There was no greater difference in the results from the Cox regression analyzes above if we included all patients without CAD (*n* = 522) and used elevated troponin (both stationary elevated and dynamic hsTnT) as covariable (Additional file 2: Table S2).

### Primary and secondary endpoints in patients with known CAD (*n* = 85)

**Fig. 3:**
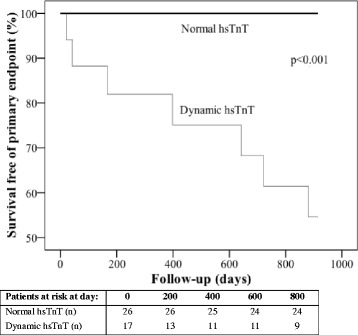
Kaplan Meier plot of survival free of primary end point in relation to normal troponin vs. dynamic hsTnT elevation in patients with known CAD. *p*-value by Breslow test, *CAD* coronary artery disease, *hsTnT* high sensitivity troponin T

Seven patients (41%) with dynamic hsTnT (*n* = 17) and no patients with normal hsTnT (*n* = 26) reached the primary endpoint during follow-up (Additional file 1: Table S1). The Kaplan Meier plot of survival free of primary endpoint in the two groups is illustrated in Fig. 3. Cox proportional hazard regression analysis was not performed because of zero events in the referent group.

Patients with dynamic hsTnT and known CAD had a higher occurrence of the primary endpoint (7 of 17 patients) compared to patients without CAD (4 of 49 patients), (*p* < 0.001).

### Dynamic hsTnT elevation and type 2 MI

Patients with known CAD had significantly higher proportion of patients with dynamic hsTnT change as compared to patients without known CAD (17 of 85 (20%) vs. 49 of 522 (9.5%); *p* = 0.003). In patients without known CAD 27 (55%) of patients with dynamic hsTnT change had chest pain and/or significant ST depression at presentation (10 with chest pain, 7 with ST depression and 10 with both) and thus, by definition, a potential type 2 MI. In the patients with known CAD and dynamic hsTnT change (*n* = 17), all had clinical evidence of acute myocardial ischemia (12 with chest pain, 5 with ST depression and 2 with both).

Multivariable logistic regression analysis (covariables that were most strongly associated with dynamic troponin elevation in the univariable analyses were included in the multivariable model) showed that higher age and the presence of chest pain or ST depressions on ECG at presentation were independently associated with dynamic hsTnT change in the analysis including all patients (Table 3). When patients with stationary elevated hsTnT were excluded (Additional file 3: Table S3) or when only patients with elevated troponin values were included in the analysis (Additional file 4: Table S4), chest pain and ST depression on ECG remained independently associated with dynamic hsTnT change.

**Table 3:** Significance of clinically relevant background factors in predicting dynamic hsTnT elevation. All patients included

	Univariable analysis		Multivariable analysis^a^	
*n* = 607	OR (95% CI)	*p*-value	OR (95% CI)	*p*-value
Male sex	0.95 (0.57–1.6)	0.85		
Age (years)	1.03 (1.01–1.06)	0.004	1.03 (1.00–1.05)	0.03
Known CAD	2.4 (1.3–4.4)	0.004	1.6 (0.82–3.0)	0.18
Hypertension	0.87 (0.52–1.5)	0.59		
Hyperlipidemia	1.2 (0.72–2.1)	0.45		
Diabetes	1.4 (0.68–2.9)	0.36		
Chest pain	3.3 (2.0–5.5)	<0.001	2.9 (1.7–5.0)	<0.001
Systolic BP (mmHg)	1.00 (0.99–1.01)	0.97		
Heart rate (beats/min)	1.01 (1.00–1.03)	0.04		
ST depression on ECG	2.4 (1.4–4.3)	0.002	2.1 (1.2–3.7)	0.01
Creatinine (μg/L)	1.00 (0.99–1.01)	0.50		

*OR* odds ratio, *BP* blood pressure, *CAD* coronary artery disease, *hsTnT* high sensitivity troponin T, *CI* confidence interval, *ECG* electrocardiography

^a^Adjustments were made for age, known CAD, chest pain and ST depression on ECG

A subanalysis showed no increased risk for the primary endpoint in non-CAD patients with dynamic hsTnT elevation and chest pain and/or ECG changes compared to non-CAD patients with dynamic hsTnT with no clinical evidence of myocardial ischemia (*p* = 0.83), all other non-CAD patients (*p* = 0.64) or non-CAD patients with normal hsTnT (*p* = 0.32).

## Discussion

### Primary analysis

Recently published review articles emphasize the need for more clinical research on dynamic hsTn elevations and type 2 MI in various clinical contexts [9, 25]. In this study we aimed to address the common and increasing clinical problem of AF patients with RVR and dynamic troponin elevations.

The key finding of this study is that we failed to demonstrate any increased risk of ACS, revascularization or death due to ischemic heart disease in patients without CAD who present with AF, RVR and dynamic troponin elevation compared to patients with normal hsTnT. But nevertheless these patients have increased all-cause mortality.

Prior research investigating the significance of acute troponin elevations in AF patients is limited. However, some of these studies have reported an association with an increased risk of MI and cardiac death during follow-up [3, 5, 26], while others could not show that elevated troponin levels predicted presence of coronary artery stenosis on angiography [2, 4]. All of these studies used older non-sensitivity troponin I assays, resulting in a smaller proportion of patients with troponin values classified as elevated and consequently higher troponin values in the “elevated troponin group”. For example, in a study by Conti et al. only 6% of the study population had elevated troponin I values (defined as >100 ng/L, 99th percentile) compared to 46% with hsTnT above the 99th percentile in our study and Gupta et al. report a mean troponin I of 560 ng/L (>10 times higher than the 99th percentile) in patients with elevated troponin compared to a mean hsTnT of 54 ng/L (approximately 4 times higher than the 99th percentile) in our study [3, 26]. This could be the reason why our results differ, since the risk of adverse outcomes has been shown to increase proportionally to troponin levels [27, 28]. Also, in clinical practice, patients with such high troponin levels often undergo further cardiac evaluation, which can be seen in the aforementioned studies [3, 5, 26]. Thus, different troponin assays make comparison with our study difficult. Other important differences between the current study and previous ones is that the previous studies included patients with known CAD, did not have a heart rate criterion for inclusion of AF patients and did not evaluate if troponin had a dynamic or non-dynamic pattern of elevation.

Our study may therefore be seen as the first to examine the significance of minor dynamic hsTnT elevations in AF patients and our results offers no greater support to our hypothesis that dynamic hsTnT elevations in the setting of AF and RVR would unmask previously unknown but clinically significant CAD. We do however acknowledge that the number of patients who reached the primary endpoint in the primary analysis were few (*n* = 16) and thus that there might be a risk that the study is underpowered to detect different event rates between groups.

According to a post hoc power calculation, where we assumed that 4% of the patients with normal hsTnT reach the primary endpoint, at a significance level of 0.05 and a power of 80%, our sample size is large enough to show a significant difference if 15% of patients with dynamic hsTnT and no known CAD would reach the primary endpoint (8.2% actually reached the primary endpoint in our study). In clinical practice we argue that it might be justifiable not to further evaluate all these patients, given the modest risk. In support of this argument a study of patients with type 2 MI showed that further cardiac investigations do not result in additional cardiac therapies or improved prognosis [10].

### Patients with known CAD

In the subgroup analysis of patients with known CAD we note that dynamic hsTnT change is associated with an increased risk of the primary endpoint during follow-up. Although the number of patients and observations are few, the trend is that our hypothesis might be true in patients with an already established CAD and that these patients might benefit from further cardiac evaluation and preventive measures. On the other hand, we find this result to be of less clinical importance as most patients with known CAD already have, or at least should have, intensive pharmacological and non-pharmacological preventive treatment and follow-up.

### Type 2 myocardial infarction

To our knowledge this is also the first study to investigate the incidence of dynamic high-sensitivity troponin elevations and possible type 2 MI in AF patients with RVR. We report the following results: Firstly, in patients without CAD presenting with AF and RVR, around 9% had dynamic hsTnT elevations and approximately half of them (5% of all non-CAD patients) could be classified as having a type 2 MI. Secondly, dynamic troponin elevation and possible type 2 MI were more common in patients with known CAD. Thirdly, the factors most strongly correlated to dynamic hsTnT change were chest pain or ST depressions on ECG at presentation even in the subgroup were patients with normal hsTnT were excluded (Additional file 4: Table S4). This observation would support the hypothesis that dynamic troponin elevation is due to acute myocardial ischemia, in contrast to stationary elevated troponin levels whose cause is more elusive [29]. However, the reason for this proposed acute myocardial ischemia is unclear. Fourthly, patients with possible type 2 MI or dynamic hsTnT elevations have roughly the same increased all-cause mortality risk as patients with stationary elevated hsTnT compared to patients with normal hsTnT.

It is important to point out that we cannot be completely sure that the observed dynamic hsTnT elevations were not caused by a type 1 mechanism as only four of the 49 patients without known CAD were further evaluated for cardiac ischemia (one with bicycle stress test and three with coronary angiography) during the index hospitalisation. However, as we previously have discussed, our results imply that the patients with dynamic hsTnT elevation did not have a markedly increased risk of the primary endpoint. We argue that this makes it less likely that the observed troponin elevations were due to an acute coronary artery plaque rupture, since so many untreated ACS most likely should have been found during follow-up. Consequently we suggest that we classify the events discussed above as type 2 myocardial infarctions.

### Study limitations

Firstly, this study was carried out only in two university hospitals in Sweden. Even though our baseline characteristics are partly comparable to other studies investigating AF patients with troponin elevations [3, 5, 16, 17, 26], our results are not necessarily generalizable to other patient populations.

Secondly, this is a retrospective study with the usual accompanying limitations and the outcomes are based on register follow-up data, which causes a degree of uncertainty to the results. However, all primary endpoints in the primary analysis were reviewed in the medical charts and all patients receiving UA or AMI as primary diagnosis underwent coronary angiography showing significant stenosis and in most cases succeeding revascularization. Thus, we can be fairly confident that all acute MIs were correctly classified as type 1 MI.

Thirdly, CAD cannot be excluded in every individual in the 522 patients without known CAD since testing was not carried out in all these patients.

Fourthly, some of our hsTnT samples were analysed during the time period when there was a problem with the calibration of the Elecsys Troponin T high sensitivity assay [30]. The analytical error was more pronounced near the LoD than the 99th percentile and we do not believe that it has significantly affected our results.

Fifthly, as previously discussed, our study might be underpowered to show a minor difference in our primary analysis, but we argue that such a small difference might not be clinical significant.

Sixthly, Because of the relatively low number of primary endpoints we were not able to adjust for all potential confounders in our cox regression analyzes. This makes our primary analysis vulnerable to confounding.

Consequently, the results of this study have to be interpreted with caution. However, despite the relatively small study size and low number of primary endpoints we believe that this is an important study that describes a significant clinical problem and that our results can be of great help in designing future studies.

Prospective studies are needed to further clarify the best strategy on how to handle AF patients with RVR and minor dynamic troponin elevations.

## Conclusions

Among AF patients without known CAD, presenting with RVR, neither patients with dynamic hsTnT elevation nor patients with stationary elevated hsTnT might not have any increased risk of ACS, revascularization or death due to ischemic heart disease compared to patients with normal hsTnT. However, elevated troponin no matter if it is stationary or dynamic is associated with increased all-cause mortality. This might suggest that the dynamic release pattern does not matter, instead it could be the elevated troponin value itself that says something about the prognosis.

Many previous studies have shown that elevated troponin values are an independent risk marker for poor prognosis in AF patients [15–18]. The reason for this is probably multifactorial and can currently only be an area of speculation. Suggested mechanisms for myocardial ischemia, necrosis, apoptosis, stress and/or dysfunction with subsequent troponin elevation are subendocardial ischemia due to shortening of diastole, myocardial stretch, left ventricular wall strain, microvascular perfusion impairment, oxidative stress, neurohormonal activation and demand-supply mismatch [9, 16, 17, 31–33].

Since the reason for dynamic troponin elevations in AF patients with RVR still remains unknown it is hard to suggest a strategy how to improve the prognosis for these patients. However, our results imply that it is unlikely that coronary events or death due to ischemic heart disease fully account for the observed adverse prognosis and that further evaluation regarding CAD may not be worthwhile in all these patients.

## Additional files

Additional file 1: Table S1. Baseline characteristics, presentation and outcomes in the study population with CAD according to troponin subgroups. (XLSX 38 kb)

Additional file 2: Table S2. Hazard ratios for the primary endpoint in all patients without CAD. (XLSX 9 kb)

Additional file 3: Table S3. Significance of clinically relevant background factors in predicting dynamic hsTnT elevation. Patients with stationary elevated hsTnT are excluded. (XLSX 10 kb)

Additional file 4: Table S4. Significance of clinically relevant background factors in prediciting dynamic hsTnT elevation. Patients with normal hsTnT values are excluded. (XLSX 10 kb)

## Abbreviations

ACS: Acute coronary syndrome
AF: Atrial fibrillation
AMI: Acute myocardial infarction
CABG: Coronary artery bypass grafting
CAD: Coronary artery disease
CHF: Chronic heart failure
CI: Confidence interval
ECG: Electrocardiography
ED: Emergency department
EF: Ejection fraction
HR: Hazard ratio
hsTnT: High-sensitivity troponin T
ICD-10: International Classification of Diseases, version 10
LoD: Limit of detection
MI: Myocardial infarction
OR: Odds ratio
PCI: Percutaneous coronary intervention
RVR: Rapid ventricular response
TIA: Transient ischemic attack
UA: unstable angina

## References

[1] Ball J, Carrington MJ, McMurray JJ, Stewart S (2013). Atrial fibrillation: profile and burden of an evolving epidemic in the 21st century. Int J Cardiol.

[2] Parwani AS, Boldt LH, Huemer M, Wutzler A, Blaschke D, Rolf S (2013). Atrial fibrillation-induced cardiac troponin I release. Int J Cardiol.

[3] Gupta K, Pillarisetti J, Biria M, Pescetto M, Abu-Salah TM, Annapureddy C (2014). Clinical utility and prognostic significance of measuring troponin I levels in patients presenting to the emergency room with atrial fibrillation. Clin Cardiol.

[4] Redfearn DP, Ratib K, Marshall HJ, Griffith MJ (2005). Supraventricular tachycardia promotes release of troponin I in patients with normal coronary arteries. Int J Cardiol.

[5] van den Bos EJ, Constantinescu AA, van Domburg RT, Akin S, Jordaens LJ, Kofflard MJ (2011). Minor elevations in troponin I are associated with mortality and adverse cardiac events in patients with atrial fibrillation. Eur Heart J.

[6] Conti A, Mariannini Y, Viviani G, Poggioni C, Cerini G, Luzzi M (2013). Abnormal troponin level as short-term predictor of poor outcome in acute atrial fibrillation. Am J Emerg Med.

[7] Mahajan VS, Jarolim P (2011). How to interpret elevated cardiac troponin levels. Circulation.

[8] Thygesen K, Mair J, Giannitsis E, Mueller C, Lindahl B, Blankenberg S (2012). How to use high-sensitivity cardiac troponins in acute cardiac care. Eur Heart J.

[9] Agewall S, Giannitsis E, Jernberg T, Katus H (2011). Troponin elevation in coronary vs. non-coronary disease. Eur Heart J.

[10] Shah AS, McAllister DA, Mills R, Lee KK, Churchhouse AM, Fleming KM (2015). Sensitive troponin assay and the classification of myocardial infarction. Am J Med.

[11] Thygesen K, Alpert JS, Jaffe AS, Simoons ML, Chaitman BR, White HD (2012). Third universal definition of myocardial infarction. J Am Coll Cardiol.

[12] Baron T, Hambraeus K, Sundstrom J, Erlinge D, Jernberg T, Lindahl B (2015). Type 2 myocardial infarction in clinical practice. Heart.

[13] Saaby L, Poulsen TS, Hosbond S, Larsen TB, Pyndt Diederichsen AC, Hallas J (2013). Classification of myocardial infarction: frequency and features of type 2 myocardial infarction. Am J Med.

[14] Saaby L, Poulsen TS, Diederichsen AC, Hosbond S, Larsen TB, Schmidt H (2014). Mortality rate in type 2 myocardial infarction: observations from an unselected hospital cohort. Am J Med.

[15] Magnoni M, Masson S, Andreini D, Moccetti T, Modena MG, Canestrari M, et al. Usefulness of high-sensitivity cardiac troponin T for the identification of outlier patients with diffuse coronary atherosclerosis and low-risk factors. Am J Cardiol. 2016; 10.1016/j.amjcard.2016.02.002 26976791

[16] Hijazi Z, Wallentin L, Siegbahn A, Andersson U, Alexander JH, Atar D (2014). High-sensitivity troponin T and risk stratification in patients with atrial fibrillation during treatment with apixaban or warfarin. J Am Coll Cardiol.

[17] Hijazi Z, Oldgren J, Andersson U, Connolly SJ, Ezekowitz MD, Hohnloser SH (2012). Cardiac biomarkers are associated with an increased risk of stroke and death in patients with atrial fibrillation: a randomized evaluation of long-term anticoagulation therapy (RE-LY) substudy. Circulation.

[18] Roldan V, Marin F, Diaz J, Gallego P, Jover E, Romera M (2012). High sensitivity cardiac troponin T and interleukin-6 predict adverse cardiovascular events and mortality in anticoagulated patients with atrial fibrillation. J Thromb Haemost.

[19] Weijs B, Pisters R, Haest RJ, Kragten JA, Joosen IA, Versteylen M (2012). Patients originally diagnosed with idiopathic atrial fibrillation more often suffer from insidious coronary artery disease compared to healthy sinus rhythm controls. Heart Rhythm.

[20] Nucifora G, Schuijf JD, van Werkhoven JM, Trines SA, Kajander S, Tops LF (2011). Relationship between obstructive coronary artery disease and abnormal stress testing in patients with paroxysmal or persistent atrial fibrillation. Int J Cardiovasc Imaging.

[21] Fletcher GF, Ades PA, Kligfield P, Arena R, Balady GJ, Bittner VA (2013). Exercise standards for testing and training: a scientific statement from the American Heart Association. Circulation.

[22] Thelin J, Borna C, Erlinge D, Ohlin B (2013). The combination of high sensitivity troponin T and copeptin facilitates early rule-out of ACS: a prospective observational study. BMC Cardiovasc Disord.

[23] Thelin J, Melander O, Ohlin B (2015). Early rule-out of acute coronary syndrome using undetectable levels of high sensitivity troponin T. European heart journal Acute cardiovascular care.

[24] Roffi M, Patrono C, Collet JP, Mueller C, Valgimigli M, Andreotti F (2016). 2015 ESC guidelines for the management of acute coronary syndromes in patients presenting without persistent ST-segment elevation: task force for the Management of Acute Coronary Syndromes in patients presenting without persistent ST-segment elevation of the European Society of Cardiology (ESC). Eur Heart J.

[25] Alpert JS, Thygesen KA, White HD, Jaffe AS (2014). Diagnostic and therapeutic implications of type 2 myocardial infarction: review and commentary. Am J Med.

[26] Conti A, Angeli E, Scorpiniti M, Alesi A, Trausi F, Lazzeretti D (2015). Coronary atherosclerosis and adverse outcomes in patients with recent-onset atrial fibrillation and troponin rise. Am J Emerg Med.

[27] Lindahl B, Venge P, James S (2010). The new high-sensitivity cardiac troponin T assay improves risk assessment in acute coronary syndromes. Am Heart J.

[28] Chew DP, Briffa TG, Alhammad NJ, Horsfall M, Zhou J, Lou PW (2015). High sensitivity-troponin elevation secondary to non-coronary diagnoses and death and recurrent myocardial infarction: an examination against criteria of causality. European heart journal Acute cardiovascular care.

[29] White HD (2011). Pathobiology of troponin elevations: do elevations occur with myocardial ischemia as well as necrosis?. J Am Coll Cardiol.

[30] Apple FS, Jaffe AS (2012). Clinical implications of a recent adjustment to the high-sensitivity cardiac troponin T assay: user beware. Clin Chem.

[31] Goette A, Bukowska A, Dobrev D, Pfeiffenberger J, Morawietz H, Strugala D (2009). Acute atrial tachyarrhythmia induces angiotensin II type 1 receptor-mediated oxidative stress and microvascular flow abnormalities in the ventricles. Eur Heart J.

[32] Range FT, Schafers M, Acil T, Schafers KP, Kies P, Paul M (2007). Impaired myocardial perfusion and perfusion reserve associated with increased coronary resistance in persistent idiopathic atrial fibrillation. Eur Heart J.

[33] Hijazi Z, Oldgren J, Siegbahn A, Granger CB, Wallentin L (2013). Biomarkers in atrial fibrillation: a clinical review. Eur Heart J.

